# HIV-Sero-prevalence trend among blood donors in North East Ethiopia

**DOI:** 10.4314/ahs.v17i3.13

**Published:** 2017-09

**Authors:** Bekele Sharew, Assefa Mulu, Brhanu Teka, Tigabu Tesfaye

**Affiliations:** 1 Department of Medical Laboratory Sciences, Wollo University, Dessie, Ethiopia P.O. Box: 1145; 2 Pharmacy department, Wollo University, Dessie, Ethiopia P. O. Box: 1145; 3 Dessie Blood Bank, Dessie, Ethiopia P. O. Box: 510

**Keywords:** Blood donor, HIV, seroprevalence, Ethiopia

## Abstract

**Background:**

Although blood transfusion is one of the known therapeutic interventions that cuts across a number of clinical disciplines. It is necessary to test all intending blood donors for HIV infection before donation. The aim of this study was to determine the prevalence of HIV among blood donors at Dessie Blood Bank, Northeast Ethiopia.

**Methods:**

A retrospective study was conducted in Dessie Blood Bank through the year 2008–2012. Sera from blood donors were tested for the detection of Anti HIV by using 4^th^ generation ELISA. Data were abstracted from records and analyzed using Microsoft Excel sheet.

**Results:**

From the total of 9384 screened blood samples collected, the prevalence of HIV in blood donors in the blood bank was 5.1% in the five consecutive years but the trend of HIV infection has decreased from 2008(5.2%) to 2012 (2.3%). The age groups 15–24 and 35–44 were the highest prevalence and the age group 45–54 was the lowest prevalence of HIV infection. The prevalence of HIV among female (7.9%) was higher than in male donors (4.4%). The trend of HIV infection was decreasing for both male and female blood donors.

**Conclusion:**

The prevalence of HIV infections among blood donors is still high in this study setting, and needs constant monitoring to evaluate prevention and control strategies to reduce the burden of transfusion-transmissible HIV infections.

## Background

Although blood transfusion is one of the known therapeutic interventions that cut across a number of clinical disciplines, the practice is not without risks. HIV has continued to create a great challenge to transfusion medicine, especially in Africa about 10–15 % of HIV transmission had been correlated with blood transfusions.^1^ Since the detection that HIV is transmitted through blood transfusion it became necessary to test all intending blood donors for HIV infection before they are transfused to ensure the safety of all blood and blood products to the recipients.^2^

Sub-Saharan Africa has the most serious HIV epidemic in the world. In 2012, about 25 million people were living with HIV accounting for nearly 70% of the global total. In the same year, there were an estimated 1.6 million new HIV infections and 1.2 million AIDS-related deaths.^3^

HIV prevalence varies significantly between regions in sub-Saharan Africa as well as individual countries. HIV prevalence in East Africa is generally moderate to high, and next to Southern Africa. However, general prevalence has been in decline for the past two decades.^3^

In 2013, there were an estimated 793,700 people living with HIV. HIV adult prevalence is anticipated to be 1.5% in 2011, the year in which the last Ethiopian Demographic Health Survey (DHS) was conducted. However prevalence varies according to age, sex, gender and geographical location.^4^

The overall seroprevalence of HIV was 3.8% in a study conducted on blood donors at Gondar university blood bank unit^5^ and the peak ages for AIDS cases were 25 to 29 for both sexes. The age range at which people become infected was 15 to 24 years for females and 25 to 34 years for males.^6^ Among blood donors in Addis Ababa the prevalence for women was 6.9% in 1999, higher than that of men.^7^

On the other hand, significantly declining fashion of HIV seroprevalence were observed in the studies done in blood banks of Jimma University Specialized Hospital and Gonder University hospital^7^,^8^ and NorthWest Ethiopia.^9^ The Federal Ministry of Health reported the National HIV prevalence as 3.5% and 5% among blood donors in 2005 where the prevalence for those blood donors in the age group 15–19 years was 2.9% but the highest prevalence occurred among donors in the age group of 30–39 years.^10^

In Nigeria, the prevalence of HIV positive donors among the screened volunteers who satisfied the criteria for blood donation was 0.87%.^11^ Whereas HIV incidences of 22% among blood donors in Kampala Uganda in East Africa which is significantly higher than the Nigerian studies.^12^

A study in India, on a rural population in 2003 reported that, the prevalence of HIV among blood donors to be 1.56%.^13^ The overall seroprevalence of HIV among the total blood donors in nation wide and in Central Blood Transfusion Service (CBTS), Kathmandu, Nepal through the six years of review (from 2001–2007) was 0.33% and 0.4% respectively.^14^

Moreover, there is limited epidemiological data on transfusion-transmissible infections (TTI) among blood donors in Ethiopia. The magnitude of HIV among blood donors has shown variation across different settings in Ethiopia that attribute to difference in societal associated risk factors and screening methods used.^15^–^19^ The safety of blood for transfusion is a major challenge of Ethiopia, and is not well addressed. As a result, use of unsafe blood products puts the patient at risk of acquiring many TTIs. Such patients can become potential sources of infection to the community that increase the disease burden, and can pose additional financial burden for diagnosis and treatment of these diseases.^20^,^21^ To ensure the safety and adequacy of the national blood supply in Ethiopia, mobilization and recruitment of low-risk, non-remunerated blood donors and testing of donated blood for TTIs using sensitive and specific testing methods are essential.^22^ Therefore, evaluating the epidemiology of TTIs can be used as indicator to assess the safety blood supply and helps to develop strategies aimed at reducing the disease burden in the communities which will ensure accessibility of safe blood supply to transfusion centers. Therefore, the aim of this study was to provide information about the trend of HIV seropositivity among the blood donors at Dessie blood bank over the study period and this would allow comparison of the seropositivity over the course of time. The finding could also be used to update intervention programs which focus on the prevention and control of HIV/AIDS.

## Methods

A retrospective study was conducted at Dessie District Blood Bank during July to September 2013 by reviewing the log book's 2008–2012 data. This was a retrospective study conducted in Dessie Blood Bank Center. The study population was all blood donors who donated blood at Dessie Blood Bank from 2008–2012. The participants were those who weighed not less than 50 kg and were age of greater than or equal to 18 years old. A total of 9384 blood donors' records were reviewed and included in the study. Serum samples were tested for HIV using fourth generation Enzyme Linked Immunosorbent Assay (ELISA) (HIV1/2: Vironostika HIV Uni-Form II AG/Ab, Bio-Merieux, Boxtel, Netherlands). All the tests were done following the manufacturer's instructions.

Data on socio-demographic variables, laboratory test results were collected from registration book of Dessie District Blood Bank using data extraction format. Data were cross-checked for completeness. The data was cleaned, edited and entered into computer and analyzed using Microsoft Excel sheet and the results were presented in tables and paragraph.

## Ethical approval

Ethical clearance was obtained from Wollo University Ethical Review Committee and verbal consent was obtained from Blood Bank administration before the commencement of data collection. Confidentiality of the information was ensured as codes instead of the names of the subjects were registered on the data collection format.

## Results

**Fig. 1:**
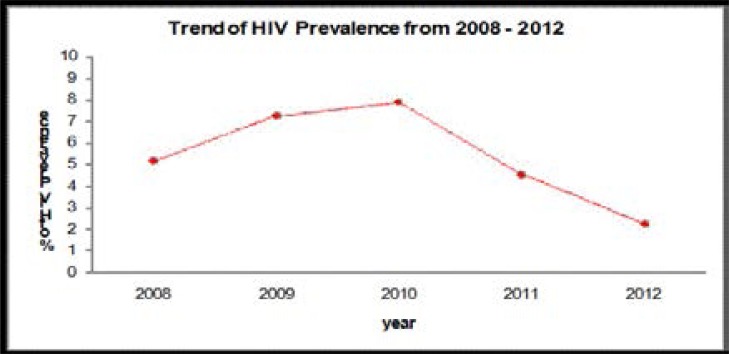
Trend of HIV prevalence among blood donors at Dessie Blood from 2008–2012

During the 5-year period, 9,384 individuals donated blood and screened for HIV infections. From the total donors, 7514 (80.1%) and 1870 (19.9%) were male and female donors respectively. More men participated in the survey than women, with a male-to-female gender ratio of 4.01. The finding of this study showed that there were 476 HIV positive blood donors. Therefore, the overall prevalence of HIV infection in blood donors was found to be 5.1% in the five consecutive years (Figure 1 and Table 1).

**Table 1:** Trend of HIV prevalence among blood donors at Dessie Blood Bank from 2008–2012

Year	No of Units screened	HIV positive	

		N	%
2008	1432	75	5.2
2009	1538	112	7.3
2010	1720	136	7.9
2011	1924	89	4.6
2012	2770	64	2.3
Total	9384	476	5.1

The trend of HIV infection has decreased from 2008 to 2012. Considering age, the age groups 15–24 and 25–34 had the highest contribution and the age group 55–65 the lowest contribution of HIV infection (Table 2 and 3).

**Table 2:** Infection with HIV among blood donors at Dessie Blood Bank by age groups from 2008 to 2012.

			Year			Total	

group	2008	2009	2010	2011	2012	N	%
15–24	33	48	66	25	26	198	41.6
25–34	19	47	46	48	30	190	40
35–44	14	13	14	15	8	64	13.4
45–54	7	2	9	1	-	19	4
55–65	2	2	1	-	-	5	1
Total	75	112	136	89	64	476	100

**Table 3:** Prevalence of HIV among age group at Dessie Blood Bank from 2008–2012.

Age	Total donors	HIV positive	Prevalence
15–24	2938	198	6.75
25–34	4355	190	4.36
35–44	1023	64	6.25
45–54	865	19	2.20
55–65	206	5	2.43
Total	9384	476	5.1

As indicated in table 4 the prevalence of HIV among female blood donors (7.9%) was higher than among male donors (4.4%). The trend of HIV infection was decreasing for both male and female blood donors.

**Table 4:** Distribution of HIV positive blood donors by gender at dessie blood bank from 2008 –2012.

Year	Total donors	Gender		HIV(+ve)	

		Male Donors	Female donors	Male N (%)	Female N (%)
2008	1432	1194	238	54 (4.5)	21(8.8)
2009	1538	1201	337	76(6.3)	36(10.7)
2010	1720	1373	347	98(7.1)	38 (11.0)
2011	1924	1502	422	61(4.1)	28(6.6)
2012	2770	2244	526	40 (1.8)	24(4.6)
Total	9384	7514	1870	329(4.4)	147 (7.9)

## Discussion

Blood transfusion is considered as a potential risk factor for transmission of viruses which are considered to be life-threatening and have a global public health importance such as HIV. In this study, the overall prevalence of HIV infection was 476 (5.1%) but showed a decreasing trend from 2008 to 2012. A decreasing trend in HIV seroprevalence among blood donors was reported from Kathmandu, Nepal^14^ and West African country, Mali.^15^

The prevalence of HIV is lower when compared with previous studies done in Ethiopia: Bahir dar, NorthWest Ethiopia^9^ and Jimma, Ethiopia^8^. It is also lower than studies done in African countries.^5^,^11^,^16^–^19^,^23^–^25^ Our study reported higher prevalence of HIV infection compared to previous studies done in Hawassa, Ethiopia (1.6%)^15^, Jijiga, Ethiopia (0.1%)^16^, Iran (0.004%)^26^, Mangalore (0.1%)^27^, Egypt (0%)^28^ and Jordan (0%)^29^. Such differences in seroprevalence rate might be due to some differences in risk behaviors, geographical variation, educational programs, preventive measures, public awareness, condition of epidemic, donor selection criteria and selection procedure, sensitivity and specificity of screening technologies employed in blood transfusion centers of those countries by performance characteristics of test kits as well as diagnostic algorithms used in each study.

Sex specific prevalence of HIV infection was 4.4% for males and 7.9% for females. The difference between the two sexes was consistent with other studies that showed a higher prevalence among females.^8^,^30^,^31^, yet the age specific prevalence was highest among the age group 15–24 years followed by those who were 25–34 years. The higher rate of seroprevalence in these age groups might be attributed to their being more sexually active.

## Conclusion

The prevalence of HIV infections among blood donors is still high in this study setting, and needs constant monitoring to evaluate prevention and control strategies. To reduce the possible risk of infections, provision of strict criterion in recruitment of blood donors by promoting the culture of voluntary blood donations, screening of blood and blood products for these pathogens using sensitive laboratory test kits are imperious. Creating community awareness about the mode of transmission and prevention of HIV infection should be strengthened by giving health education. Moreover, conducting further community-based studies to identify societal risk factors exposing communities for blood-borne infections and developing population-specific interventions to interrupt transmission are valuable in recruiting potential volunteer non-remunerated blood donors.
